# Effect of Blood Orange (*Citrus sinensis* L. Osbeck) Peel Waste as a Feed Additive on the Growth Performance, Digestive Enzyme Activity, Antioxidant Capacity, and Immune Response in Juvenile Black Rockfish (*Sebastes schlegelii*)

**DOI:** 10.3390/antiox13121452

**Published:** 2024-11-26

**Authors:** Tae Hoon Lee, Ki-Tae Kim, Hwa Yong Oh, Seo Young Park, Gyu Jin Lee, Hyun-Soo Kim, Hee Sung Kim

**Affiliations:** 1 Department of Marine Biology and Aquaculture, Gyeongsang National University, Tongyeong 53064, Republic of Korea; dlxogns1204@naver.com (T.H.L.); oho1203@gnu.ac.kr (H.Y.O.); emily010628@naver.com (S.Y.P.); hn5511@naver.com (G.J.L.); 2 Southeast Sea Fisheries Research Institute, National Institute of Fisheries Science, Tongyeong 53017, Republic of Korea; oysterkim@korea.kr; 3 Department of Seafood Science and Technology, Gyeongsang National University, Tongyeong 53064, Republic of Korea; gustn783@gnu.ac.kr

**Keywords:** *Citrus sinensis* (L.) Osbeck peel, growth, antioxidant response, immune capacity, *Sebastes schlegelii*

## Abstract

This study evaluated bioactive compounds in blood orange (*Citrus sinensis* (L.) Osbeck) peel (BOP) as dietary additives. An 8-week feeding trial was conducted to investigate the effects of dietary supplementation on the growth performance, body composition, digestive enzyme activity, antioxidant capacity, and immune response of juvenile black rockfish. A total of 1260 juvenile rockfish (1.4 ± 0.01 g) were randomly distributed into seven treatment groups, each with 50 fish per circular tank. The groups were fed seven different diets containing graded levels of 0 (control, BOP0), 1 (BOP1), 2 (BOP2), 3 (BOP3), 5 (BOP5), 7 (BOP7), and 10 (BOP10) g kg^−1^, respectively. The BOP10 diet significantly enhanced the final weight, weight gain, specific growth rate, protein efficiency ratio, and protein retention in fish. The BOP treatments notably affected the fishes’ whole-body crude protein and lipid contents. Plasma total cholesterol levels of fish fed the BOP0 and BOP1 diets were significantly higher than those fed the BOP7 and BOP10 diets. The activities of trypsin and lipase were significantly affected by dietary BOP levels. The antioxidant enzyme activity in the plasma of fish fed the BOP10 diet was significantly higher than those fed the BOP0 diet. The lysozyme activity and levels of immunoglobulin M and G in fish fed the BOP0 diet were significantly lower than those in fish fed the BOP10 diet. In conclusion, dietary supplementation of BOP at 10 g kg^−1^ improved the growth performance and overall health of juvenile black rockfish.

## 1. Introduction

Recently, numerous activities have been directed toward contributing to the United Nations Sustainable Development Goals (UN-SDGs) and environmental conservation through the global shift toward a “circular economy” for resource sustainability. This necessitates bolstering the international role by offering solutions to global challenges [1,2]. Extensive studies have concentrated on maintaining the ongoing sustainability of resources in aquaculture. One such approach involves the use of plant by-products as ingredients in fish feed.

Plant by-products primarily originate from vegetables and fruits used in the production of various items such as juice, jam, and canned food [3,4]. These plant by-products, derived from reliable food sources, do not harbor bacterial, fungal, or parasitic diseases that could indirectly affect human health [5]. Numerous fruit by-products are extensively utilized in aquafeed as a source of functional feed additives [6,7,8,9]. In particular, the use of fruit peel by-products, which are predominantly discarded at present, is anticipated to result in cost savings in the disposal process of plant by-products and provide an alternative source for feed additives. Several studies have demonstrated the use of various fruit residues, specifically peel, as dietary supplements in fish diets. Peels of fruits such as apple, banana, sweet orange, mango, and pineapple have been fed to various cultured fish such as genetically improved farmed tilapia (GIFT, *Oreochromis niloticus*) [10], Rohu (*Labeo rohita*) [11], and *O. niloticus* [12]. Moreover, lemon peels have been utilized as additives for gilthead seabream (*Sparus aurata* L.), Nile tilapia (*O. niloticus*), and African catfish (*Clarias gariepinus*) diets [13,14]. All of the aforementioned sources of peel have demonstrated beneficial effects in terms of growth and health performance in several farmed fish.

Blood oranges (*Citrus sinensis* L. Osbeck), also known as pigmented sweet oranges or red oranges, originated from a spontaneous bud mutation [15]. The three main cultivars of blood orange, namely “Tarocco”, “Moro”, and “Sanguinelli”, are considered the most important citrus varieties in southern Italy [16]. Blood oranges are widely cultivated in the Mediterranean basin, particularly in Spain and Italy, and also in Asian countries. The global annual production of blood oranges is approximately 79 million tons [17]. Consumer interest in blood orange fruit has grown due to the antioxidant activity of anthocyanin and other bioactive compounds it contains, including ascorbic acid (vitamin C, VC), phenolics, and flavonoids [18]. Owing to its health benefits, which include protective substances against cancer, arteriosclerosis, and heart disease, the blood orange fruit is considered to be the best citrus fruit [19]. Annually, around 30 million tons of citrus fruits are processed globally for juice extraction [20]. However, this juice production results in significant amounts of residue, primarily consisting of the peels [21]. Therefore, blood orange peel (BOP), a waste residue produced during the blood orange juice processing stage, is readily available and cost-effective for use as a feed additive. BOP is rich in phenolic compounds, flavonoids (such as anthocyanins), and other nutrients [22]. These substances exhibit considerable antioxidant, antimicrobial, and antibacterial properties [23,24,25]. Despite the bioactive properties of BOP, there is a lack of studies evaluating the effects of its inclusion in aquatic animal diets on overall health.

The black rockfish (*Sebastes schlegelii*) holds significant importance in aquaculture in East Asia, including China, Japan, and Korea. In 2022, the culture production of black rockfish reached 16,189 tons [26], making it the second highest in Korea. However, due to increased demand, black rockfish have been cultured using high-density practices, leading to heightened stress that negatively impacts their growth performance and immune systems. Functional feed additives that are environmentally sustainable, cost-effective, easily accessible, and derived from natural sources present a potential solution to these challenges in current rockfish culture. However, there is a lack of information regarding the benefits of dietary BOP for this species. Therefore, this study aimed to assess the bioactive compounds in BOP used as dietary additives and investigate their effect on the growth performance, digestive enzyme activity, antioxidant capacity, and immune response of juvenile black rockfish.

## 2. Materials and Methods

### 2.1. Preparation of BOP and Chemical Analysis

Fresh Moro blood oranges were purchased from a local fruit store in Seogwipo-si, Jeju-do, Republic of Korea. The oranges were meticulously peeled, ensuring complete separation of the edible sections. The extracted peels were washed and dried using an agricultural product dryer (KED-M07D1, Kiturami Co. Ltd., Seoul, Republic of Korea) at 20 °C. They were then ground using a kitchen blender. The resulting powdered form of BOP was stored at 4 °C for subsequent use.

The flavonoid content of BOP was measured using the method outlined by Moreno et al. [27]. A 1 mL sample was diluted with 4.3 mL of 80% ethanol, 0.1 mL of 10% aluminum nitrate, and 0.1 mL of 1 mol L^−1^ aqueous potassium acetate. After a 40 min incubation at room temperature in a dark environment, the absorbance at 415 nm was measured. Quercetin (Sigma-Aldrich Co., St. Louis, MO, USA) was used as a standard.

The concentration of VC in BOP was determined using high-performance liquid chromatography (Agilent 1200 Series HPLC; Agilents Technologies, Anaheim, CA, USA) with an ultraviolet (UV) detector set at 254 nm. A 0.05 M KH_2_PO_4_ solution was used as the mobile phase at a pH of 2.8 with a flow rate of 1.0 mL/min. Manually weighed samples were homogenized in 10% cooling metaphosphoric acid. The homogenates were centrifuged at 3000× *g* for 20 min. The supernatants were subjected to HPLC analysis after filtration through a 0.45 μm-pore-size syringe filter (Sartorius, Gottingen, Germany).

DPPH scavenging activity was analyzed using the Blois [28] method. For the positive control, 80 μL of the sample or ascorbic acid was mixed with 100 μL of 150 μM DPPH in methanol. The mixture was left undisturbed for 10 min at room temperature. The absorbance was measured at 525 nanometers using a microplate reader (SpectraMax^®^ M2/M2e, Sunnyvale, CA, USA).

The radical-scavenging activity of ABTS was assessed using the method outlined by Re et al. [29]. The ABTS radical was produced by exposing 7 mM ABTS to 2.4 mM potassium persulfate in a light-free environment for 16 h. Subsequently, the working solution was prepared by diluting the ABTS to achieve an absorbance of 1.5 at 414 nm with distilled water. The absorbance was measured at 414 nm by mixing 100 µL of the working solution with 50 µL of the sample or ascorbic acid as the positive control. The mixture was left undisturbed for 5 min at room temperature.

### 2.2. Formulation of Experimental Diets

In this study, seven isonitrogenous and isolipidic experimental diets were formulated (Table 1). These diets contained seven different levels of BOP: 0 g kg^−1^ diet (BOP0, control), 1 g kg^−1^ diet (BOP1), 2 g kg^−1^ diet (BOP2), 3 g kg^−1^ diet (BOP3), 5 g kg^−1^ diet (BOP5), 7 g kg^−1^ diet (BOP7), and 10 g kg^−1^ diet (BOP10). The dry feed ingredients were thoroughly mixed to ensure homogeneity. Then, fish and soybean oils, along with distilled water, were added to the mixture to form a dough. The dough was chopped using a chopper (3.0 mm diameter, SL Machinery, Incheon, Republic of Korea) to form pellets of 3.0 mm diameter. The pellets were then dried at a temperature of 20 °C using an agricultural product dryer (KED-M07D1, Kiturami Co. Ltd., Seoul, Republic of Korea) for two days. The experimental diets were stored at −20 °C until use. All of the experimental diets met the VC level of juvenile rockfish, as per [30].

### 2.3. Feeding Trial Condition and Design Experiment

Juvenile *S. schlegelii* were sourced from a commercial hatchery situated in Namhae-gun, Gyeongsangnam-do, Republic of Korea, and subsequently relocated to the Marine Bio-Education and Research Center at Gyeongsang National University in Tongyeong, Gyeongsangnam-do, Korea. Prior to the feeding trial, the fish were acclimated to the experimental conditions and fed commercial extruded pellets (Jeil Feed Co., Haman, Gyeongsangnam-do, Republic of Korea) comprising 52% crude protein and 10% crude lipids.

Subsequently, a cohort of 1260 juvenile rockfish, each with an average weight of 1.4 ± 0.01 g, were randomly allocated to 21 circular tanks, each with a total volume of 300 L. The fish were housed at a density of 60 individuals per tank, and a flow-through system with a water flow rate of 2.7 L/min was established. Over a period of 8 weeks, the five experimental diets were manually administered to triplicate groups of fish at 09:00 and 17:00 h daily. Excess feed and feces were removed via siphoning. Water quality parameters, including dissolved oxygen (6.50 ± 0.41 mg L^−1^), salinity (31.8 ± 0.37 psu), and temperature (21.2 ± 0.71 °C), were periodically monitored and found to be within the acceptable range.

### 2.4. Growth Performance Parameters

Following a day of fasting at the conclusion of the feeding trial, the fish were anesthetized using MS-222 (Sigma-Aldrich, Saint Louis, MO, USA) at a concentration of 150 ppm prior to sampling. The body weight and total length of all the fish in each tank were measured to calculate growth performance parameters (growth and feed utilization) using the subsequent formula.

Survival (SR, %) = (number of fish at the end of the trial/number of fish at the beginning of the trial) × 100

Weight gain (WG) = [(final body weight–initial body weight)/initial body weight]

Specific growth rate (SGR, %/day) = [ln final weight of fish–ln initial weight of fish]/days of feeding × 100

Feed consumption (FC, g/fish) = total dry feed intake/fish

Feed efficiency (FE) = WG of fish/feed consumed

Protein efficiency ratio (PER) = WG of fish/protein consumed

Protein retention (PR, %) = protein gain × 100/protein consumed

Condition factor (CF) = fish weight × 100/total length3

Hepatosomatic index (HSI, %) = 100 × (hepatopancreas weight/whole-body weight)

Viscerosomatic index (VSI, %) = 100 × (viscera weight/whole-body weight)

### 2.5. Proximate Body Composition

After the eight-week feeding trial, ten fish were randomly selected from each tank and anesthetized with MS-222. For chemical composition analysis, each body was processed into a finely chopped and homogenized paste. The crude protein content (N × 6.25 mm) was measured using a KD310–A–1015 KjelROC Analyzer (OPSIS Liquid LINE, Furulund, Sweden) following the Kjeldahl digestion process. The crude lipid content was determined using the Soxhlet extraction method and a Soxtec extractor (ST 243 Soxtec^TM^; FOSS, Hillerod, Denmark). To measure moisture and ash contents, the samples were dried in an oven at 105 °C for 24 h and then in a muffle furnace at 600 °C for 4 h.

### 2.6. Digestive Enzyme Analysis

Intestinal samples from ten fish in each tank were homogenized in 10 volumes (*v*/*w*) of ice-cold, 0.86% physiological saline using a TissueLyser II (QIAGEN, Venlo, The Netherlands) in an ice bath. The samples were subsequently centrifuged at 13,000 rpm for 10 min at 4 °C. The supernatant obtained was used to assess the enzymatic activity of amylase, trypsin, and lipase using a commercially available kit (Abcam, Cambridge, UK). The unit of measurement for intestinal enzymatic activity is moles per minute (U) per milligram of protein (U mg protein^−1^). The protein concentration was determined according to Bradford [31] using bovine serum albumin (Sigma, Saint Louis, MO, USA) as a standard.

### 2.7. Biochemical Analyses and Antioxidant Enzyme Activity

Blood samples were collected from the caudal veins of ten anesthetized fish in each tank. Plasma was obtained by centrifuging the samples at 4 °C for 15 min at 7000 rpm. The plasma samples were stored at −80 °C for subsequent analysis of biochemical indices and antioxidant enzyme activity.

Plasma biochemical parameters, such as aspartate aminotransferase (AST), alanine aminotransferase (ALT), total cholesterol (TCHO), total protein (TP), and glucose (GLU), were measured using an automated chemistry system, the Fuji Dri-Chem NX500i, manufactured by Fujifilm in Tokyo, Japan.

The activities of superoxide dismutase (SOD), catalase (CAT), and glutathione (GSH) content were measured in the plasma samples as per the instructions provided in the manual of the commercial kit (Cayman’s Assay Kit, Cayman Chemical, Ann Arbor, MI, USA). The absorbance was evaluated using a spectrophotometer (Thermo Scientific MULTISKAN GO, Vantaa, Finland).

### 2.8. Immunological Analysis

The serum lysozyme activity was ascertained using a turbidimetric assay, as outlined in the study by Lange et al. [32]. Briefly, a 0.05 M sodium phosphate buffer (pH 6.2) was used to dissolve a 1.9 mL suspension of *Micrococcus lysodeikticus* (0.2 mg/mL; Sigma, St. Louis, MO, USA), to which 100 μL of test serum was added. The chemical reactions were carried out at a temperature of 25 °C, and the absorbance at 530 nm was measured using a spectrophotometer (Thermo Fisher Scientific, Tewksbury, MA, USA) for a duration of 0 to 60 min. The quantity of enzyme needed to induce a 0.001/min decrease in absorbance was used to calculate the lysozyme activity unit. The concentrations of immunoglobulin (Ig) M and G in plasma were measured by enzyme-linked immunosorbent assay (ELISA) quantification kits (MyBioSource Inc., San Diego, CA, USA).

### 2.9. Statistical Analyses

Before analysis, all percentage values underwent an arcsine transformation. The results were reported as the mean value plus or minus the standard error (SE). Levene’s test was used to evaluate the homogeneity of variances among treatments. Subsequently, the data were subjected to a one-way analysis of variance (ANOVA) and examined using Tukey’s HSD test to identify significant differences (*p* < 0.05). The statistical analyses were performed using the SPSS version 27.0 program (SPSS Inc., based in Chicago, IL, USA).

## 3. Results

### 3.1. Chemical Composition and Antioxidant Activities of BOP

The concentrations of VC, total phenolics, and total flavonoids in BOP were found to be 4.38 mg kg^−1^, 28.4 gallic acid mg 100 g^−1^, 14.3 mg quercetin g^−1^, and 364.8 mg g^−1^, respectively (Table 2). BOP demonstrated an inhibitory effect on free-radical production, as evidenced by the DPPH and ABTS radical-scavenging activities (%) under the assay conditions and concentrations used (Table 2).

### 3.2. Growth Performance

Table 3 presents the effects of varying dietary levels of BOP on growth, feed utilization, and organosomatic indices. The final weight, WG, and SGR of fish fed the BOP10 diet were significantly higher (*p* < 0.05) than those of fish fed the BOP0 diet. However, no significant differences were observed among the other treatments (*p* > 0.05). The PER of fish fed the BOP10 diet was significantly higher (*p* < 0.05) than that of fish fed the BOP0 and BOP3 diets, but it did not significantly differ from that of fish fed the BOP1, BOP2, BOP5, and BOP7 diets (*p* > 0.05). The PR in fish fed the BOP10 diet was significantly higher than that of fish fed the BOP0 and BOP1 diets (*p* < 0.05). However, no significant changes were observed in the FE, CF, VSI, or HSI among fish fed the experimental diets (*p* > 0.05).

### 3.3. Whole-Body Composition

Table 4 illustrates the effects of dietary BOP on the whole-body composition of fish. The crude protein content in the whole bodies of fish fed the BOP7 and BOP10 diets was significantly higher than that of fish fed the BOP0, BOP1, BOP2, and BOP3 diets (*p* < 0.05). However, this difference was not significant when compared to fish fed the BOP5 diet (*p* > 0.05). Fish fed the BOP10 diet exhibited a significantly higher whole-body crude lipid content than that of fish fed the BOP0, BOP1, BOP2, and BOP3 diets (*p* < 0.05), but this was not significantly different when compared to fish on the BOP5 diet (*p* > 0.05). Nevertheless, the dietary BOP did not significantly affect the moisture and ash contents of the whole bodies of juvenile black rockfish (*p* > 0.05).

### 3.4. Hematological Indices

The hematological parameters of juvenile black rockfish are outlined in Table 5. The concentration of TCHO in the plasma of fish fed the BOP0 and BOP1 diets was significantly higher than that of fish fed the BOP7 and BOP10 diets (*p* < 0.05). However, it did not significantly differ from that of fish fed the BOP2, BOP3, and BOP5 diets (*p* > 0.05). Notably, the levels of AST, ALS, GLU, and TP did not vary significantly (*p* > 0.05) between the BOP0 diet and other treatment groups.

### 3.5. Digestive Enzyme Activities

Table 6 presents the effects of varying dietary levels of BOP on the activity of intestinal digestive enzymes in juvenile black rockfish. The trypsin activity in the intestines of fish fed the BOP10 diet was significantly higher than that of fish fed the BOP0 diet (*p* < 0.05), but it did not significantly differ from that of fish fed the BOP1, BOP2, BOP3, BOP5, and BOP7 diets (*p* > 0.05). The lipase activity was significantly higher in the intestines of fish fed the BOP10 diet compared to those fed the BOP0, BOP1, BOP2, BOP3, and BOP5 diets (*p* < 0.05), but it did not significantly differ from that of fish fed the BOP7 diet (*p* > 0.05). In contrast, the activity of amylase in the intestines of fish did not significantly differ across the experimental treatments (*p* > 0.05).

### 3.6. Antioxidant Enzyme Activities

Table 7 illustrates the effects of varying dietary levels of BOP on the antioxidant enzyme activity in the plasma of rockfish. The activities of SOD and CAT in the plasma of fish fed the BOP10 diet were significantly higher than those of fish fed the BOP0 diet (*p* < 0.05). However, these activities did not significantly differ from those of fish fed the BOP1, BOP2, BOP3, BOP5, and BOP7 diets (*p* > 0.05). The plasma GSH content of fish fed the BOP7 and BOP10 diets was significantly higher than that of fish fed the BOP0 diet (*p* < 0.05), but it did not significantly differ from that of fish fed the BOP1, BOP2, BOP3, and BOP5 diets (*p* > 0.05).

### 3.7. Immunological Parameters

Table 8 presents the immunological parameters of juvenile black rockfish for each treatment. The serum lysozyme activity in fish fed the BOP0 diet was significantly lower than that of fish fed the BOP2, BOP3, BOP5, BOP7, and BOP10 diets (*p* < 0.05). The levels of IgM and IgG in the serum of fish fed the BOP10 diet were significantly higher than those of fish fed the BOP0 diet (*p* < 0.05).

## 4. Discussion

Numerous studies have demonstrated that incorporating fruit peels into aquaculture feeds at optimal levels as feed additives can yield positive results in terms of fish growth, feed utilization, antioxidant capacity, and immune responses [11,33,34]. The use of fruit peels, often discarded as by-products, in commercial aquaculture could significantly contribute to global efforts to establish environmentally sustainable and eco-friendly aquaculture practices. When added to the diet of farmed fish, the peels of various fruits, such as sweet oranges, pomegranates, bananas, pineapples, watermelon, etc., have been suggested to enhance growth and overall health [11,12,34,35,36]. It is anticipated that blood orange (*C. sinensis* L. Osbeck) peels may produce similar effects in fish when combined with other fruit peels. However, research on their use as an aquaculture feed additive is currently limited. Previous research has explored the impact of different types of citrus fruit peels on growth, hematological properties, immune system response, antioxidant capacity, and digestive parameters across multiple fish species [13,35,37,38]. Therefore, the aim of the present study was to evaluate the content and antioxidant activity of residual functional substances in BOP and to determine their dietary supplementation effects on the growth and various health parameters of juvenile black rockfish.

Citrus peels are inherently rich in a variety of phytochemical compounds, which exhibit significant antioxidant, antibacterial, and antimicrobial properties [24,39]. This study found that the levels of VC, flavonoids, and phenolics in BOP were in line with the results obtained by Ghasemi et al. [40], who compared the chemical composition of peels from 13 different citrus species. The substantial presence of VC, flavonoids, and phenolics in citrus peels suggests that these peels may have stronger antioxidant and antibacterial effects compared to other parts of the fruit [41,42]. Furthermore, the antioxidant activity of citrus fruit peels appears to stimulate the cellular antioxidant enzymes in aquatic organisms [43,44].

Growth enhancement is considered a critical characteristic in aquaculture, directly influencing the profitability of the farmer and the productivity of the aquaculture system [45]. Our findings suggest that supplementing the rockfish diet with 10 g kg^−1^ BOP significantly increased weight gain (WG), SGR, PER, and PR values compared to the control group (BOP0). The growth rate, WG, SGR, and PER of *Catla catla* were significantly influenced by sweet orange (*C. sinensis*) peel extracts at concentrations ranging from 2 to 6 g kg^−1^, as reported by Shabana et al. [46]. The inclusion of bergamot (*C. bergamia*) peel oil in the diets of Nile tilapia and European sea bass (*Dicentrarchus labrax*) improved their growth performance [47,48]. Similarly, Samavat et al. [43] observed that supplementing Caspian white fish (*Rutilus kutum*) diets with varying concentrations of grapefruit (*C. paradis*) peel extract enhanced fish growth performance. Moreover, this study observed a significant increase in intestinal trypsin and lipase activities when BOP was added to the diet at a concentration of 10 g kg^−1^. The improved WG and SGR of the fish fed with BOP at a dose of 10 g kg^−1^ in their diet could be attributed to the enhanced activity of trypsin and lipase enzymes. Our investigation revealed a beneficial association between beneficial biologically active substances, increased growth performance, and digestive enzyme activities [49]. In this context, dietary VC enhanced growth performance and feed utilization by improving intestinal enzyme activity, morphology, digestion, and absorption [50]. Additionally, plant phenolic compounds can promote the growth of beneficial gut bacteria, functioning like prebiotics [51]. This phenomenon may be attributed to certain phytochemicals providing absorbents capable of binding to and eliminating pathogens and undesirable components in the digestive tract, thereby promoting the absorption of essential nutrients [52,53].

The study found that fish fed a diet supplemented with BOP10 exhibited higher protein levels and decreased lipid levels throughout their bodies compared to the control group fed a BOP0 diet. These observations could be linked to improved protein utilization, as evidenced by higher PER and PR values, decreased amino acid digestion for energy, and increased lipid accumulation in body tissues [54]. Furthermore, plant-based polyphenol compounds are recognized for their fat-reducing and antioxidant properties [55,56]. Notably, there were significant differences in the PER and PR between the BOP10 and BOP0 groups. In a similar vein, the inclusion of sweet orange peel in the diets of gilthead seabream led to a simultaneous increase in whole-body protein content and a decrease in lipid content [57]. Moreover, the oral administration of green tea (*Camellia sinensis*) leaves, known for their rich flavonoid content, resulted in an increase in the protein content in the whole body of Nile tilapia [58]. In this study, it is plausible to hypothesize that the observed higher protein content in the BOP10 group resulted from lipid catabolism, which conserves protein and energy. To ascertain whether enhanced digestibility leads to improved growth performance and body composition, further research is needed to explore the impact of BOP on nutrient digestibility.

Blood biochemistry serves as a valuable tool for assessing the physiological and health status of aquatic animals [59,60]. The effects of supplementing fish diets with citrus fruit peels in in vitro feeding experiments have been well documented, particularly in terms of blood biochemistry [38,47,61]. Citrus fruit peels notably contain flavonoids (polymethoxylated flavones and hesperitin) that have been demonstrated to lower cholesterol levels in animal studies [42,62]. Similarly, in this study, the addition of more than 7.0 g kg^−1^ BOP effectively lowered TCHO levels in fish. Furthermore, the serum GLU, globulin, TCHO, triglyceride, ALT, and AST levels in Nile tilapia were reduced when astringent lemon peel was administered at concentrations of 7.5 and 10.0 g kg^−1^ diet [38]. Additionally, Toutou et al. [47] reported that the serum glucose levels of Nile tilapia and thin-lip mullet (*Liza ramada*) significantly decreased when lime peel was included in diets supplemented with vitamin-C-rich lemon peel. From the results of this study, it was found that diets supplemented with BOP did not significantly affect AST, ALT, GLU, or TP levels, indicating that the BOP additive does not adversely affect the hematological health of juvenile black rockfish.

Digestive enzymes play a crucial role in facilitating the digestion and absorption of nutrients in organisms, and their activity can serve as an indicator of the degree of feed digestion and absorption [63]. The evaluation of intestinal digestive enzyme activity in fish can indirectly reflect the species’ capability to digest feed [64]. In this study, the impact of a diet supplemented with 10 g kg^−1^ BOP on nutrient absorption and metabolism was assessed by examining the activity of intestinal digestive enzymes, namely trypsin and lipase. Research has suggested that plant by-products can enhance the activities of digestive enzymes such as lipase, trypsin, alkaline phosphatase, protease, and amylase, thereby facilitating the digestion and absorption of lipids, carbohydrates, and proteins [65,66,67]. Specifically, Liu et al. [68] posited that the ability of VC to act as an extracellular reactive oxygen species scavenger in vivo, thereby protecting the hepatopancreas from lipid peroxidation, contributes to the enhancement of intestinal digestive enzyme activity. This enzyme activity indirectly influences the assimilation capacity in fish, which is vital for nutrient digestion and assimilation. Furthermore, assimilation is linked with intestinal cell maturation, development, and body weight gain, thereby promoting nutrient supply. Additionally, the antibacterial properties of polyphenols enable beneficial microorganisms to degrade nutrients through the secretion of digestive enzymes [69,70]. The findings of this study provide further evidence supporting the idea that VC and phenolic compounds present in BOP might enhance the digestive system of juvenile rockfish, thereby facilitating their nutrient uptake.

The antioxidant enzyme system in animals, primarily composed of SOD, CAT, glutathione reductase, and glutathione peroxidase, protects cells against free radicals and facilitates their elimination through the antioxidant defense mechanism [71]. This study found that the plasma antioxidant capacity (SOD, CAT, and GSH) of juvenile rockfish was enhanced in response to a diet supplemented with 10 g kg^−1^ BOP. Similar trends in the elevation of SOD and CAT levels were observed in the European sea bass in response to bergamot peel oil [48]. Additionally, significant increases in CAT and SOD enzyme activities were reported in Nile tilapia and African catfish provided with lemon (*C. limon*) peels at doses of 10 and 20 g kg^−1^ in the diet [14]. Furthermore, levels of SOD, CAT, and glutathione peroxidase increased in common carp (*Cyprinus carpio*) fed a mixture of *Bacillus licheniformis* and lemon peel powder [72]. Similarly, Rohu showed a significant increase in the levels of SOD, CAT, and glutathione peroxidase when administered diets containing 2.5 and 5 g kg^−1^ dried lemon peel. Polyphenols are widely recognized for their antioxidant properties, including the ability to scavenge free radicals and inhibit the production of oxygen anions [73,74]. The presence and quantity of phenolic rings, which neutralize free radicals, including peroxyl radicals, anion superoxides, and hydroxyl radicals, are associated with antioxidant properties [75]. Moreover, flavonoids, by protecting antioxidant defenses and upregulating intracellular signaling associated with the antioxidant cellular response, are thought to be capable of modulating cellular responses to various stimuli [53].VC also exhibits the capacity to lower free radicals and stimulate glutathione peroxidase activity [76]. In this study, the presence of bioactive compounds in BOP, including phenolic compounds, flavonoids, and VC, may have contributed to the enhancement of the antioxidant status of the fish. This aligns with the findings of the authors of [77], who discovered that these compounds in sweet orange peel facilitated the elimination of superoxide radicals by increasing the secretion of antioxidant enzymes.

Lysozyme activity, which triggers the lysis of pathogenic bacteria, is a crucial defense mechanism [78]. In fish, the immunoglobulins IgM and IgG play a vital role in enhancing innate and adaptive immunity by activating complement pathways and facilitating cellular cytotoxicity [79]. This study found that lysozyme, IgM, and IgG levels were significantly elevated in groups fed diets containing 10 g kg^−1^ of BOP. This aligns with previous findings where feeding Nile tilapia and African catfish with lemon peel significantly increased lysozyme activity [14]. Similarly, the lysozyme activity of common carp was enhanced when they were fed a combination of *Bacillus licheniformis* and lemon peel [72]. Furthermore, Rohu, when given 2.5 and 5 g kg^−1^ of dried lemon peel in their diet, showed increased activity of the alternative complement pathway and elevated levels of lysozyme and IgM [37]. Beltrán et al. [13] observed that both the humoral (IgM) and cellular immunity of gilthead seabream, along with the expression of immune-related genes, were enhanced by the dietary addition of 15 and 30 g kg^−1^ dehydrated lemon peel. While there is limited research examining the impact of citrus fruit peels on the immune response of fish and even less is known about the specific mechanisms by which these products influence aquatic organisms, these results suggest that the beneficial bioactive compounds in citrus fruit peels are partially responsible for these enhancements [80,81,82]. Interestingly, phenolic compounds are known to undergo intestinal absorption and interact with the intestinal immune system, thereby inducing a protective response in the host [83]. Ding et al. [83] also reported that a wide variety of cell types contain receptors for polyphenols, which can activate signaling pathways and elicit an immune response. However, further studies are needed to establish the exact mechanisms of the observed immunostimulatory effects of bioactive substances in BOP and whether they are related to defense against disease through immune enhancement.

## 5. Conclusions

In conclusion, based on the findings of this study, a diet for juvenile black rockfish supplemented with BOP at a concentration of 10 g kg^−1^ could serve as an ideal growth and health stimulant. BOP is seen as a beneficial dietary additive that can enhance growth performance, antioxidant activity, and immune response. Based on the findings, 10 g kg^−1^ BOP supplementation to the diet of juvenile rockfish was the most beneficial approach for enhancing overall health status.

## Figures and Tables

**Table 1:** Detailed composition and proximate analysis of the experimental diets supplemented with varying levels of blood orange peel (BOP), expressed as g kg^−1^ in dry matter.

Ingredients	Experimental Diets						
	BOP0	BOP1	BOP2	BOP3	BOP5	BOP7	BOP10
Sardine meal	580	580	580	580	580	580	580
Dehulled soybean meal	95	95	95	95	95	95	95
Wheat flour	220	219	218	217	215	213	210
BOP	0	1	2	3	5	7	10
Fish oil	40	40	40	40	40	40	40
Soybean oil	40	40	40	40	40	40	40
Vitamin premix ^1^	10	10	10	10	10	10	10
Mineral premix ^2^	10	10	10	10	10	10	10
Choline	5	5	5	5	5	5	5
Proximate composition (g kg^−1^, dry matter basis)							
Dry matter	91.2	90.8	90.9	90.9	90.8	90.3	90.3
Crude protein	522	517	516	515	516	516	517
Crude lipid	140	138	136	140	142	140	139
Ash	98	99	97	97	100	102	98
Vitamin C (mg kg^−1^)	120.2	125.7	130.2	133.6	140.7	148.6	161.0

^1^ Vitamin premix contained the following amount, which was diluted in cellulose (g kg^−1^ mix): L-ascorbic acid, 121.2; DL-α-tocopheryl acetate, 18.8; thiamin hydrochloride, 2.7; riboflavin, 9.1; pyridoxine hydrochloride, 1.8; niacin, 36.4; Ca-D-pantothenate, 12.7; myo-inositol, 181.8; D-biotin, 0.27; folic acid, 0.68; p-aminobenzoic acid, 18.2; menadione, 1.8; retinyl acetate, 0.73; cholecalciferol, 0.003; cyanocobalamin, 0.003. ^2^ Mineral premix contained the following ingredients (g kg^−1^ mix): MgSO_4_·7H_2_O, 80.0; NaH_2_PO_4_·2H_2_O, 370.0; KCl, 130.0; ferric citrate, 40.0; ZnSO_4_·7H_2_O, 20.0; Ca-lactate, 356.5; CuCl, 0.2; AlCl_3_·6H_2_O, 0.15; KI, 0.15; Na_2_Se_2_O_3_, 0.01; MnSO_4_·H_2_O, 2.0; CoCl_2_·6H_2_O, 1.0.

**Table 2:** Quantitative analysis of vitamin C, total phenolic compounds, flavonoids, and anthocyanin contents, along with the radical-scavenging activities of the ethanol extract derived from blood orange peel (BOP).

	BOP Composition							
Chemical compounds	Vitamin C (mg kg^−1^)	4.38 ± 0.48						
	Total phenolics (gallic acid mg 100 g^−1^)	28.4 ± 3.34						
	Total flavonoids (quercetin mg g^−1^)	14.3 ± 3.80						
Radical-scavenging activities	Concentration (µg mL^−1^)	4000	2000	1000	500	250	125	IC_50_
	DPPH (%)	57.0 ± 0.52	38.9 ± 0.50	30.0 ± 0.69	27.9 ± 0.81	23.1 ± 0.43	20.8 ± 0.53	5.5
	ABTS (%)	67.5 ± 0.63	57.4 ± 0.72	32.9 ± 0.31	21.4 ± 0.68	13.1 ± 0.96	7.0 ± 0.69	5.5

Abbreviations: BOP, blood orange peel; DPPH, 1,1–diphenyl–2–picrylhydrazyl; ABTS, 2,2′–azinobis–(3–ethylbenzothiazoline–6–sulfonate).

**Table 3:** Evaluation of growth performance over an 8-week period in juvenile black rockfish fed experimental diets supplemented with different levels of BOP.

Parameters	Experimental Diets							*p*-Value
	BOP0	BOP1	BOP2	BOP3	BOP5	BOP7	BOP10	
Initial weight (g/fish)	1.4 ± 0.00	1.4 ± 0.00	1.4 ± 0.00	1.4 ± 0.00	1.4 ± 0.00	1.4 ± 0.00	1.4 ± 0.00	-
Final weight (g/fish)	12.9 ± 0.05 ^a^	12.9 ± 0.12 ^a^	13.0 ± 0.11 ^ab^	13.1 ± 0.09 ^ab^	13.1 ± 0.16 ^ab^	13.1± 0.08 ^ab^	13.4 ± 0.11 ^b^	0.035
SR (%)	100.0 ± 0.00 ^a^	100.0 ± 0.00 ^a^	100.0 ± 0.00 ^a^	98.9 ± 1.11 ^a^	100.0 ± 0.00 ^a^	99.4 ± 0.56 ^a^	99.4 ± 0.56 ^a^	0.642
WG (g/fish)	11.5 ± 0.05 ^a^	11.5 ± 0.12 ^ab^	11.6 ± 0.11 ^ab^	11.7 ± 0.09 ^ab^	11.7 ± 0.16 ^ab^	11.7 ± 0.08 ^ab^	12.0 ± 0.11 ^b^	0.049
SGR (%)	3.96 ± 0.007 ^a^	3.98 ± 0.015 ^a^	3.99 ± 0.015 ^ab^	4.01 ± 0.013 ^ab^	4.01 ± 0.020 ^ab^	4.01 ± 0.011 ^ab^	4.05 ± 0.016 ^b^	0.011
FI	12.0 ± 0.07 ^a^	12.0 ± 0.08 ^a^	12.1 ± 0.08 ^a^	12.4 ± 0.25 ^a^	12.2 ± 0.11 ^a^	12.3 ± 0.12 ^a^	12.4 ± 0.07 ^a^	0.244
FE	0.95 ± 0.003 ^a^	0.96 ± 0.004 ^a^	0.96 ± 0.007 ^a^	0.96 ± 0.008 ^a^	0.96 ± 0.002 ^a^	0.96 ± 0.008 ^a^	0.97 ± 0.008 ^a^	0.777
PER	1.82 ± 0.003 ^a^	1.87 ± 0.001 ^ab^	1.86 ± 0.015 ^ab^	1.83 ± 0.025 ^a^	1.87 ± 0.011 ^ab^	1.85 ± 0.007 ^ab^	1.85 ± 0.022 ^b^	0.034
PR	31.1 ± 0.21 ^a^	31.4 ± 0.12 ^a^	31.6 ± 0.47 ^ab^	32.3 ± 0.41 ^ab^	32.4 ± 0.05 ^ab^	32.5 ± 0.63 ^ab^	33.2 ± 0.09 ^b^	0.013
CF	1.88 ± 0.019 ^a^	1.88 ± 0.065 ^a^	1.86 ± 0.022 ^a^	1.88 ± 0.009 ^a^	1.84 ± 0.013 ^a^	1.83 ± 0.033 ^a^	1.87 ± 0.009 ^a^	0.859
VSI (%)	10.8 ± 0.06 ^a^	10.9 ± 0.04 ^a^	11.0 ± 0.03 ^a^	11.0 ± 0.04 ^a^	11.0 ± 0.05 ^a^	11.0 ± 0.05 ^a^	11.0 ± 0.06 ^a^	0.768
HSI (%)	3.9 ± 0.11 ^a^	3.9 ± 0.02 ^a^	3.8 ± 0.03 ^a^	3.9 ± 0.13 ^a^	3.9 ± 0.01 ^a^	3.9 ± 0.04 ^a^	3.9 ± 0.05 ^a^	0.998

Values represent means ± SE (*n* = 3). Values with different superscript letters within a row indicate significant differences (*p* < 0.05), while mean values in the same row without any superscript are not different. Abbreviations: SR, survival; WG, weight gain; SGR, specific growth rate; FI, feed intake; FE, feed efficiency; PER, protein efficiency ratio; PR, protein retention; CF, condition factor; VSI, viscerosomatic index; HSI, hepatosomatic index; BOP, blood orange peel.

**Table 4:** Proximate composition (%) of juvenile black rockfish after an 8-week period of being fed experimental diets supplemented with varying levels of BOP.

Composition	Experimental Diets							*p*-Value
	BOP0	BOP1	BOP2	BOP3	BOP5	BOP7	BOP10	
Moisture	68.5 ± 0.14 ^a^	68.6 ± 0.18 ^a^	68.4 ± 0.07 ^a^	68.5 ± 0.02 ^a^	68.4 ± 0.11 ^a^	68.6 ± 0.17 ^a^	68.1 ± 0.11 ^a^	0.155
Crude protein	17.1 ± 0.12 ^ab^	17.0 ± 0.01 ^a^	17.1 ± 0.13 ^ab^	17.4 ± 0.04 ^ab^	17.4 ± 0.10 ^bc^	17.5 ± 0.30 ^c^	17.6 ± 0.10 ^c^	0.022
Crude lipid	10.62 ± 0.083 ^c^	10.55 ± 0.023 ^bc^	10.56 ± 0.066 ^bc^	10.51 ± 0.066 ^bc^	10.43 ± 0.067 ^abc^	10.23 ± 0.035 ^ab^	10.20 ± 0.082 ^a^	0.002
Ash	3.1 ± 0.07 ^a^	3.3 ± 0.11 ^a^	3.2 ± 0.13 ^a^	3.2 ± 0.12 ^a^	3.0 ± 0.07 ^a^	3.0 ± 0.06 ^a^	3.1 ± 0.03 ^a^	0.226

Values represent means ± SE (*n* = 3). Values with different superscript letters within a row indicate significant differences (*p* < 0.05), while mean values in the same row without any superscript are not different. Abbreviation: BOP, blood orange peel.

**Table 5:** Analysis of hematological parameters in juvenile black rockfish after an 8-week period of being fed experimental diets supplemented with varying levels of BOP.

Parameters	Experimental Diets							*p*-Value
	BOP0	BOP1	BOP2	BOP3	BOP5	BOP7	BOP10	
AST (U/L)	152.0 ± 8.39 ^a^	124.3 ± 9.40 ^a^	126.3 ± 4.63 ^a^	126.0 ± 10.50 ^a^	127.3 ± 6.64 ^abc^	128.0 ± 5.29 ^a^	121.7 ± 1.86 ^a^	0.136
ALT (U/L)	35.7 ± 4.81 ^a^	33.7 ± 5.78 ^a^	30.3 ± 2.67 ^a^	39.3 ± 3.84 ^a^	33.0 ± 5.20 ^a^	28.0 ± 2.08 ^a^	31.3 ± 3.76 ^a^	0.601
TCHO (mg/dL)	232.7 ± 9.24 ^c^	226.3 ± 5.17 ^c^	221.3 ± 12.31 ^bc^	209.3 ± 5.78 ^abc^	210.7 ± 6.36 ^abc^	180.0 ± 8.19 ^a^	181.7 ± 8.82 ^ab^	0.002
GLU (mg/dL)	53.3 ± 2.85 ^a^	55.0 ± 4.00 ^a^	51.0 ± 3.61 ^a^	50.7 ± 3.28 ^a^	55.7 ± 2.73 ^a^	51.3 ± 1.67 ^a^	50.7 ± 5.24 ^a^	0.885
TP (g/dL)	6.0 ± 0.21 ^a^	6.7 ± 0.85 ^a^	5.2 ± 0.21 ^a^	5.9 ± 0.23 ^a^	5.4 ± 0.30 ^a^	4.93 ± 0.19 ^a^	5.03 ± 0.41 ^a^	0.078

Values represent means ± SE (*n* = 3). Values with different superscript letters within a row indicate significant differences (*p* < 0.05), while mean values in the same row without any superscript are not different. Abbreviations: AST, aspartate aminotransferase; ALT, alanine aminotransferase; TCHO, total cholesterol; GLU, glucose; TP, total protein; BOP, blood orange peel.

**Table 6:** Assessment of digestive enzyme activities (mU mg^−1^ protein) in juvenile rockfish after an 8-week period of being fed experimental diets supplemented with varying levels of BOP.

Parameters	Experimental Diets							*p*-Value
	BOP0	BOP1	BOP2	BOP3	BOP5	BOP7	BOP10	
Amylase	60.6 ± 8.85 ^a^	66.9 ± 2.78 ^a^	72.7 ± 8.74 ^a^	76.0 ± 4.44 ^a^	78.7 ± 8.49 ^a^	84.9 ± 7.08 ^a^	91.9 ± 0.99 ^a^	0.068
Trypsin	21.1 ± 1.98 ^a^	26.8 ± 3.52 ^ab^	28.0 ± 3.50 ^ab^	28.2 ± 3.79 ^ab^	30.0 ± 4.09 ^ab^	30.8 ± 3.53 ^ab^	38.2 ± 2.33 ^b^	0.048
Lipase	32.0 ± 3.33 ^a^	45.5 ± 5.96 ^ab^	46.1 ± 2.29 ^ab^	49.7 ± 9.22 ^ab^	51.9 ± 7.38 ^ab^	77.2 ± 7.58 ^bc^	97.8 ± 7.97 ^c^	0.002

Values represent means from triplicated groups of fish where the values in the same column sharing the different superscript letter are significantly different (*p* < 0.05). Abbreviation: BOP, blood orange peel.

**Table 7:** Evaluation of plasma antioxidant enzyme activity in juvenile rockfish after an 8-week period of being fed experimental diets supplemented with varying levels of BOP.

Parameters	Experimental Diets							*p*-Value
	BOP0	BOP1	BOP2	BOP3	BOP5	BOP7	BOP10	
SOD (U/mL)	29.0 ± 2.18 ^a^	31.5 ± 2.34 ^ab^	30.7 ± 1.22 ^ab^	32.3 ± 0.81 ^ab^	33.9 ± 1.38 ^ab^	34.0 ± 0.97 ^ab^	37.2 ± 0.94 ^b^	0.039
CAT (nmol/min/mL)	648.0 ± 25.08 ^a^	653.1 ± 24.94 ^ab^	688.2 ± 14.05 ^ab^	731.1 ± 27.54 ^ab^	720.2 ± 12.84 ^ab^	725.6 ± 22.28 ^ab^	750.1 ± 13.76 ^b^	0.022
GSH (µM)	1.91 ± 0.015 ^a^	2.00 ± 0.056 ^ab^	2.01 ± 0.074 ^ab^	2.03 ± 0.050 ^ab^	2.12 ± 0.041 ^ab^	2.15 ± 0.129 ^ab^	2.32 ± 0.095 ^b^	0.037

Values represent means from triplicated groups of fish where the values in the same column sharing the different superscript letter are significantly different (*p* < 0.05). Abbreviations: SOD, superoxide dismutase; CAT, catalase; GSH, glutathione; BOP, blood orange peel.

**Table 8:** Analysis of immunological response in juvenile rockfish after an 8-week period of being fed experimental diets supplemented with varying levels of BOP.

Parameters	Experimental Diets							*p*-Value
	BOP0	BOP1	BOP2	BOP3	BOP5	BOP7	BOP10	
Lysozyme activity (U/mL)	1.14 ± 0.220 ^a^	1.76 ± 0.180 ^ab^	1.84 ± 0.135 ^b^	1.98 ± 0.059 ^b^	1.85 ± 0.082 ^b^	1.87 ± 0.092 ^b^	1.95 ± 0.014 ^b^	0.007
IgM (mg/mL)	178.0 ± 8.56 ^a^	189.4 ± 3.09 ^ab^	188.7 ± 2.93 ^ab^	191.1 ± 3.08 ^ab^	200.6 ± 5.52 ^ab^	194.9 ± 5.24 ^ab^	211.8 ± 5.60 ^b^	0.014
IgG (mg/mL)	19.7 ± 0.26 ^a^	19.9 ± 0.28 ^ab^	20.3 ± 0.43 ^ab^	21.2 ± 0.54 ^ab^	21.1 ± 0.51 ^ab^	21.4 ± 0.78 ^ab^	22.7 ± 0.62 ^b^	0.015

Values represent means from triplicated groups of fish where the values in the same column sharing the different superscript letter are significantly different (*p* < 0.05). Abbreviations: IgM, immunoglobulin M; IgG, immunoglobulin G; BOP, blood orange peel.

## Data Availability

Data available on reasonable request.
